# Transcriptome and Metabolomics Analysis Reveal the Effects of Red and Blue Light on the Physiology and Primary Medicinal Components (Liquiritin and Glycyrrhizic Acid) of *Glycyrrhiza uralensis* Seedlings

**DOI:** 10.3390/ijms26104641

**Published:** 2025-05-13

**Authors:** Yuan Jiang, Zhengru Zhang, Shurui Zhang, Xinying Chen, Baoshan Li, Siyu Ma, Yanjun Wang, Zhirong Sun

**Affiliations:** School of Chinese Materia Medica, Beijing University of Chinese Medicine, Beijing 102488, China; 2022094131@bucm.ac.cn (Y.J.); 20210935142@bucm.ac.cn (Z.Z.); 20230935161@bucm.ac.cn (S.Z.); 20240935179@bucm.ac.cn (X.C.); 20230935262@bucm.ac.cn (B.L.); 20230935160@bucm.ac.cn (S.M.); 20220935153@bucm.ac.cn (Y.W.)

**Keywords:** *Glycyrrhiza uralensis*, red–blue light, transcriptome, metablisome

## Abstract

*Glycyrrhiza uralensis* Fisch. is considered one of the most economically important medicinal plants worldwide. However, the quality of cultivated *G. uralensis* has not been adequate to meet the market demand. As one of the most important factors for plant growth, light influences the production and accumulation of metabolites in plants. However, the effect of light on the development and accumulation of components of *G. uralensis* is unclear. In this study, we found that red light and 4R1B (red/blue = 4:1) could promote the growth of licorice, such as the plant height, diameter of the reed head, and biomass accumulation, while blue light inhibited indicators of reed head diameter, biomass accumulation, etc. The impact of the light system is reflected in blue light significantly suppressing the photosynthetic rate and stomatal conductance, while red light and mixed light had the opposite effects. The red group had the lowest superoxide dismutase (SOD) activity and malondialdehyde (MDA) content, which suggested the production and scavenging of O_2_ was balanced in red light. Additionally, the red group had the highest content of soluble sugars and soluble proteins. We combined metabolomic and transcriptomic analysis and found that the gene expression in the treatment groups was up-regulated in the liquiritin synthesis pathway, and the liquiritin content of the 4R1B group and R group was significantly increased by 275% and 191% that of the CK group. Moreover, 4R1B significantly promoted the accumulation of glycyrrhizic acid (94% higher than in the CK group) and the expression of genes in the glycyrrhizic acid synthesis pathway. In addition, the light treatments affected seven phytohormone pathways (abscisic acid, brassinosteroid, salicylic acid, auxin, gibberellin, cytokinin, and jasmonic acid) in *G. uralensis*, which was related to cell elongation, stem elongation, stress resistance, and other aspects. In general, we analyzed the response mechanism of *G. uralensis* to red and blue light at the physiological, medicinal component, and molecular levels. The results will provide a new perspective for studying the regulatory effect of light quality on the growth and medicinal components of *G. uralensis*.

## 1. Introduction

*Glycyrrhiza uralensis* Fisch., commonly known as licorice, is one of the ancient herbal plants of the legume family, which is a high-value medicinal plant worldwide [1]. The roots and rhizomes of licorice have long been used as a herbal medicine and natural sweetener [2], and also employed as a flavoring additive in the food and tobacco industries [3]. The triterpenoid saponin compound glycyrrhizic acid in licorice has antiviral activity, and flavonoid compounds such as liquiritin have pharmacological activities such as antioxidant, blood lowering, and lipid-lowering activities [4,5,6]. Unfortunately, due to excessive excavation and habitat destruction in recent years, the wild resources of glycyrrhiza are increasingly scarce, and wild licorice has been listed as a second-level key protected plant in China. Artificially cultivated licorice is the main source of licorice medicinal products on the market [7,8]. Studies have found that the glycyrrhizic acid content of some cultivated licorice cannot meet the standards specified in the 2020 edition of the Pharmacopoeia of the People’s Republic of China (CHP) [9,10,11]. This resulted in artificially cultivated licorice still being unable to replace wild licorice. Therefore, how to improve the quality of cultivated *G. uralensis* is a key issue in ensuring its clinical efficacy and sustainable resource utilization.

As one of the most important environmental factors for plant growth, light is not only the basic energy of photosynthesis but also an important source of external signals [12,13]. Changes in spectra alter plant morphology and photosynthesis [14], which in turn affects the biomass accumulation [15]. For example, blue light induces larger fruits, while in *Chrysanthemum indicum*, *Stevia rebaudian*, and *Tripterospermum japonicum* blue light suppresses the stem length [16,17,18]. Blue light increases the chlorophyll a/b ratio and promotes the rate of photosynthesis per unit of leaf area [19]. Red light increases the chlorophyll content, thereby maximizing the photosynthetic capacity of *Boehmeria nivea*, and significantly increases the shoot and leaf biomass, plant height, number of leaves per plant, and stem diameter [20]. Moreover, changes in the spectrum can regulate gene expression, which in turn affects the biosynthetic accumulation of metabolites. Previous reports revealed that blue light stimulates the expression of metabolite synthesis gene such as *Dihydroflavonol-4-reductase* (*DFR*), *Chalcone synthase* (*CHS*), and *Phenylalanine ammonia-lyase* (*PAL*), thus affecting the synthesis of phenolic acids, flavonoids, and some other compounds [21,22,23]. Zhang et al. [24] found that combined blue and red light could enhance the accumulation of phenolic acids in *Salvia miltiorrhiza* by up-regulating *SmPAL1* and *Sm4CL1* transcription. Our previous research showed that compared to monochromatic light and red/blue (1:2) light, red/blue (2:1) light can promote the accumulation of glycyrrhetinic acid and glycyrrhizin in *G. uralensis*. However, the potential mechanisms through which red-to-blue light promotes growth and secondary metabolite accumulation in *G. uralensis* are still unknown.

In this study, we combined UPLC-MS/MS-based metabolomics and transcriptomics analysis to investigate the differences in gene expression and components of metabolism between *G. uralensis* grown in red light, blue light, combination lighting, and conventional white LED light. This study provides a certain theoretical and experimental basis for artificially regulating the growth and accumulation of the main medicinal components of *G. uralensis* in the future.

## 2. Results and Discussion

### 2.1. Effects of Light on the Growth and Biomass of G. uralensis

**Figure 1:**
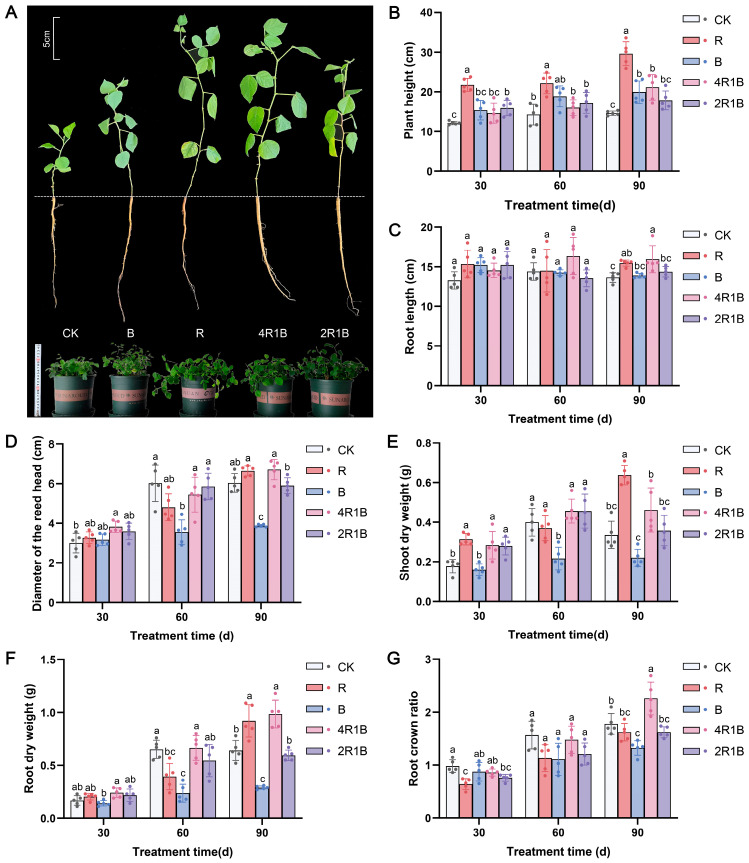
The morphology and growth index of *G. uralensis* under different light treatments. R and B mean red light and blue light. 4R1B and 2R1B indicate that the spectral proportions of red and blue light are 4:1 and 2:1, respectively. CK means white light. (**A**) is the morphology at 30 days. The top left plotting scale was used to estimate the length of a single seedling and the bottom left ruler was used to estimate the size of a potted plant. (**B**) is the plant height at 30–90 days, (**C**) is the root length at 30–90 days, (**D**) is the diameter of the reed head at 30–90 days, (**E**) is the shoot dry weight at 30–90 days, (**F**) is the root dry weight at 30–90 days, and (**G**) is the root crown ratio of *G. uralensis* at 30–90 days. The data presented are mean values (±standard error) of three replicates (*n* = 5). Values in the same column not sharing the same letter are significantly different at *p* < 0.05. R and B mean red light and blue light. 4R1B and 2R1B indicate the spectral proportion of red and blue light are 4:1 and 2:1, respectively. CK means white light.

The growth of *G. uralensis* was strongly influenced by different lights (Figure 1A). Our data showed that each indicator increased over time. The plant height, shoot dry weight, and root dry weight of the group treated with red light were significantly greater than those in the other treatment groups (Figure 1B,E,F). Compared with the CK group, the R group showed significantly increased, by 103.1%, 84.5%, and 47.2%, plant height, shoot dry weight, and root dry weight in 90 d. However, the blue monochromatic light significantly inhibited *G. uralensis* seedling growth, such as the diameter of the reed head, shoot dry weight, root dry weight, and root crown ratio. Regarding mixed light, 4R1B contains a higher proportion of red light, which is beneficial for various growth indicators compared to 2R1B. The results in Figure 1 showed that the root length, reed head diameter, root dry weight, and root-to-shoot ratio of group 4R1B were significantly greater than those in the other groups except the red light group.

The above results indicate that monochromatic red and red–blue composite light (4R1B) enhance the morphology and promote the growth of *G. uralensis*, whereas monochromatic blue light inhibits the growth of *G. uralensis*. This may be due to red light increasing the accumulation of gibberellin and being conducive to the elongation and growth of plant cells, while blue light suppresses cell division and extension growth [25]. A similar phenomenon was observed in *Salvia miltiorrhiza*, *Paris polyphylla* var. *yunnanensis*, and *Gynostemma pentaphyllum*, where red light promotes shoot growth, thereby increasing biomass, while blue light inhibits plant growth [24,26,27]. Moreover, compared with monochromatic light, red–blue composite light increases the dry weight of *Perilla frutescens*, *Ocimum basilicum*, and *Piper nigrum* [28].

### 2.2. Effects of Light Quality Treatment on the Photosynthetic Rate of G. uralensis

**Figure 2:**
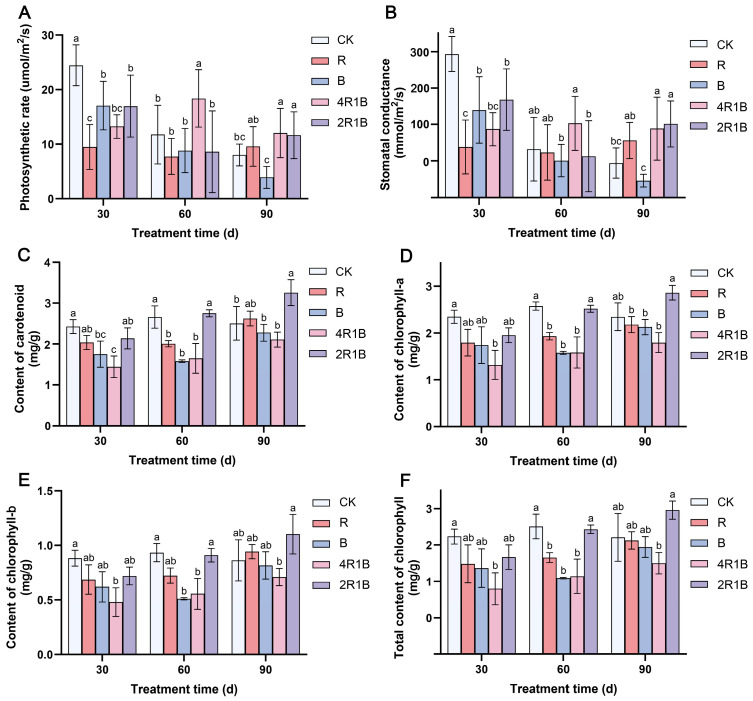
The photosynthetic characteristics of *G. uralensis* after different light treatments. (**A**) photosynthetic rate, (**B**) stomatal conductance, (**C**) content of carotenoids, (**D**) content of chlorophyll-a, (**E**) content of chlorophyll-b, and (**F**) total content of chlorophyll. The data presented are mean values (±standard error) of three replicates (*n* = 3). Values in the same column not sharing the same letter are significantly different at *p* < 0.05.

The photosynthetic characteristic parameters can reflect the photosynthetic capacity of plants. The effects of different lights on the photosynthetic rate and stomatal conductance of *G. uralensis* seedlings were similar (Figure 2A,B). At 30 d, the photosynthetic rate and stomatal conductance were significantly inhibited in all light treatment groups. This may be because monochromatic red light often leads to red light syndrome, manifested as hindered development of the photosystem, thereby severely reducing the photosynthetic rate of plants [29,30]. Among the groups, the R group and the 4R1B group had the lowest photosynthetic rate and stomatal conductance. At 60 days, the *G. uralensis* seedlings in the 4R1B group showed a significantly increased photosynthetic rate, which was 1.56 and 1.53 times higher than that of the CK group. At 90 days, mixed light groups 4R1B and 2R1B had significantly increased photosynthetic rates. This was probably because the blue and red light combination more effectively excited photoreceptors, resulting in a higher net photosynthetic rate than either monochromatic red or blue light [25,31].

The effects of different light treatments on the contents of chlorophyll-a (Figure 2C), chlorophyll-b (Figure 2D), total chlorophyll (Figure 2E), and carotenoids (Figure 2F) of *G. uralensis* seedlings were similar, and the changing trends across the different times were also consistent. During the light treatment period, the content of each photosynthetic pigment increased with the treatment time. At 30 days, the photosynthetic pigment content of the seedlings in all the light treatment groups was lower than that in the CK group. Among the seedlings, the 4R1B group seedlings contained the lowest amount of photosynthetic pigments (68–84% lower than in the CK group). At 60 days, the contents of various photosynthetic pigments increased in the 2R1B group and were not significantly different from those in the CK group. For 90 days, the content of each photosynthetic pigment in the light treatment group gradually increased, and the content of the 2R1B group increased significantly, being higher than that of the CK group.

### 2.3. Determination of SOD and MDA

**Figure 3:**
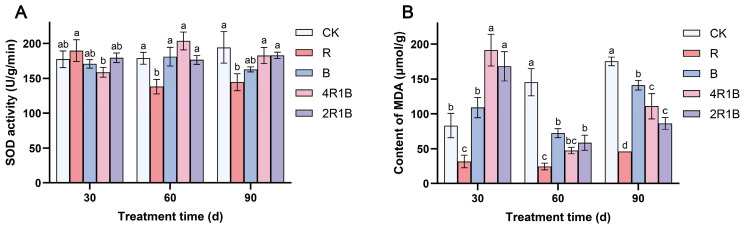
Effect of combined red and blue light on the antioxidant system of *G. uralensis*. (**A**) is SOD, (**B**) is MDA. The data presented are mean values (±standard error) of three replicates (*n* = 3). Values in the same column not sharing the same letter are significantly different at *p* < 0.05.

Figure 3A shows that during the treatment period, SOD activity increased slowly in the CK group, decreased and then slightly increased in treatment R, increased and then decreased in treatments B and 4R1B, and did not change significantly in treatment 2R1B. At 30 d, the SOD activity was highest in treatment R and lowest in treatment 4R1B, which showed significant differences from each other but not from the CK group. At 60 d and 90 d, the SOD activity of the R group was significantly lower than that of CK the group by 22.8% and 25.7%, respectively.

During the treatment period, the MDA contents increased in the CK group and decreased and then increased in the rest of the treatments (Figure 3B). At 30 d, the MDA contents in the mixed light treatments reached their highest levels, which were significantly higher than those in the CK group by 130.0% and 102.3%. The MDA content of treatment R was significantly lower than that of the CK group. At 60 d, the MDA contents in treatments R, B, 4R1B, and 2R1B were significantly higher than those in the CK group by 83.2%, 50.3%, 67.4%, and 59.7%, respectively. At 90 d, the MDA contents in the monochromatic light treatments reached their highest levels, and the MDA contents in treatments R, B, 4R1B, and 2R1B were significantly lower than those in the CK group by 73.6%, 19.5%, 36.8% and 50.8%, respectively. Treatment R reduced the MDA content in *G. uralensis* at 30–60 days.

SOD and MDA are notably influenced by light intensity in different ways [32]. Manivannan et al. [33] reported that blue light improves antioxidant enzyme activities in Rehmannia glutinosa cultured in vitro. Conversely, the antioxidant activity of pea and *Dendrobium officinale* seedlings was improved under red light [15,34]. However, in the present study, red light improved the antioxidant enzyme activities in *G. uralensis*. Moreover, *G. uralensis* under blue and mixed light conditions maintained higher production of MDA, indicating oxidative damage to lipid membranes, while RL facilitated a reduction in oxidative stress in *G. uralensis*. Analogously, Li et al. [35] reported that blue light increased membrane damage, and red light or an appropriate red–blue composite light reduced membrane damage in *Lycoris longituba* seedlings. Therefore, red light enhanced antioxidant enzyme activity in *G. uralensis* leaves, removing reactive oxygen species. These findings suggest that the light spectrum distribution evokes diverse antioxidative responses in different plant species, with contrasting results.

Reactive O_2_ species (ROS) are produced in both unstressed and stressed cells. The SODs constitute the first line of defense against ROS [36]. Manivannan et al. [37] reported that blue light improves the antioxidant enzyme activities in *Rehmannia glutinosa* cultured in vitro. Conversely, the antioxidant activity of pea and Dendrobium officinale seedlings was improved under red light [15,34]. However, in the present study, SOD activity was lowest under red light in *G. uralensis*, which suggests that under red light, the formation and removal of O_2_ are in balance. Moreover, *G. uralensis* under blue and mixed light conditions maintained higher production of MDA, indicating oxidative damage to lipid membranes, while red light facilitated a reduction in oxidative stress in *G. uralensis*. Analogously, Li et al. [35] reported that blue light increased membrane damage, and red light or an appropriate red–blue composite light reduced membrane damage in *Lycoris longituba* seedlings. Therefore, red light enhanced antioxidant enzyme activity in *G. uralensis* leaves, removing reactive oxygen species. These findings suggest that blue light may enhance antioxidant responses, while red light can alleviate the membrane damage in plants caused by blue light and help balance the production and scavenging of O_2_.

### 2.4. Determination of Total Protein and Soluble Sugar

**Figure 4:**
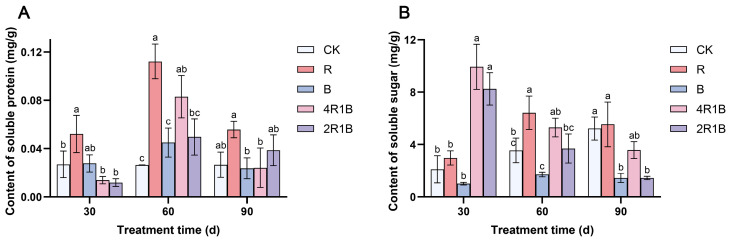
Contents of soluble protein (**A**) and soluble sugar (**B**) in *G. uralensis* under different light treatments. The data presented are mean values (±standard error) of three replicates (*n* = 3). Values in the same column not sharing the same letter are significantly different at *p* < 0.05.

During the treatment period, the soluble sugar content of the CK group showed an upward trend (Figure 4A). The soluble sugar contents of the monochromatic light treatments increased and then decreased, reaching a maximum at 60 d, and those of the mixed light treatments decreased. At 30 d, the soluble sugar contents of treatments 4R1B and 2R1B were significantly higher than those of the CK group by 371.1% and 291.3%, respectively. At 60 d, treatment R had the highest soluble sugar content, which was significantly higher than that of the CK group by 81.0%. Treatment B had the lowest soluble sugar content, showing a significant difference from treatment R but no significant difference from the CK group. At 90 d, the soluble sugar contents of treatments B and 2R1B were significantly lower than those of the CK group by 72.4% and 72.5%. Treatment R was conducive to improving the soluble sugar content of *G. uralensis* within 30–60 d.

The soluble protein (Figure 4B) content of the CK group did not change significantly with treatment time, while that of the rest of the treatments increased and then decreased and reached the maximum at 60 d. At 30 d, the soluble protein content of treatment R was significantly higher than that of the CK group by 93.5%. At 60 d, the soluble protein contents of treatments R and 4R1B were significantly higher than those of the CK group by 323.4% and 213.3%, respectively. At 90 d, there was no significant difference between the CK group and the treatments. Treatments R and B were conducive to improving the soluble sugar content of *G. uralensis* within 30–60 d.

Studies have shown that light has an impact on the nutrient contents of vegetables and fruits, such as soluble sugar and total protein, but the effects were inconsistent in various species [38,39,40]. This study showed that red light promotes the synthesis of soluble sugars and soluble proteins. Blue light obviously inhibited soluble sugar accumulation while there was no significant difference in soluble protein. Moreover, various ratios of red and blue light obviously promoted the accumulation of soluble sugar in the early seedling stage, which is consistent with the findings of the study on *Toona sinensis* conducted by Ding et al. [41].

### 2.5. Analysis of Metabolomics Differences in G. uralensis Under Different Light Treatments

**Figure 5:**
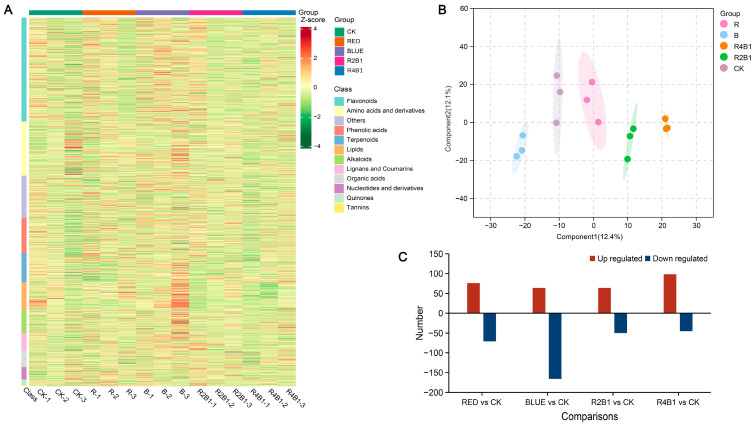
The compounds of *G. uralensis* under different light treatments. (**A**) Differential compounds accumulation in *G. uralensis*. (**B**) OPLS-DA of all samples. Each dot represents a sample with different colors representing different groups, and the circles represent the 95% confidence intervals of the samples. (**C**) Differential accumulation of compounds in different light treatments.

To further explore how different light treatments impact metabolites associated with *G. uralensis*, the roots of plants from different treatment groups were analyzed using targeted metabolomics. Differentially accumulated metabolites (DAMs) were identified according to the criterion of |log2FC| ≥ 1. In the samples treated with different lights, a total of 477 DAMs were identified, mainly including 146 flavonoids, 108 amino acids and their derivatives, 38 phenolic acids, 37 alkaloids, 32 other metabolites, 32 lipids, and 27 terpenes. The metabolite heatmap is shown in Figure 5A. The orthogonal partial least discriminant squares (OPLS-DA) result demonstrated that the metabolites of different light treatment groups were quite varied, revealing that the metabolomics data were highly reproducible and dependable (Figure 5B).

In RED vs. CK, the following DAMs (76 up- and 71 down-regulated) were identified: 41 amino acids and derivatives, 13 phenolic acids, 39 flavonoids, two lignans and coumarins, nine alkaloids, seven terpenoids, two organic acids, and 83 other metabolites (Table S1A). The following DAMs (64 up- and 166 down-regulated) were identified in the BLUE vs. CK comparison: 48 amino acids and derivatives, 18 phenolic acids, 78 flavonoids, 18 lignans and coumarins, 13 alkaloids, 12 terpenoids, 8 organic acids, and 35 other metabolites (Table S1B). For 2R1B vs. CK, the following DAMs (50 up- and 44 down-regulated) were identified: 13 amino acids and derivatives, 11 phenolic acids, 27 flavonoids, six lignans and coumarins, seven alkaloids, two terpenoids, four organic acids, and 24 other metabolites (Table S1C). Moreover, in 4R1B vs. CK, the following DAMs (98 up- and 45 down-regulated) were identified: 48 amino acids and derivatives, eight phenolic acids, 36 flavonoids, three lignans and coumarins, 21 alkaloids, eight terpenoids, four organic acids, and 15 other metabolites (Table S1D).

### 2.6. Analysis of Transcriptomics Differences in G. uralensis Under Different Light Treatments

As shown in Table S2, the transcriptome of each sample obtained an average of 741.17 million raw reads and 719.92 million clean reads after removing the linker sequence, low-quality bases, and N-containing reads. After comparing clean reads with the Trinity assembly reference sequence, a total of 666.24 million mapped reads were obtained, with an alignment rate of 91.6–93.18%. The percentages of Q30 and GC were 93.2–93.79% and 44.86–45.37%, respectively, indicating that the quality of the transcriptome sequencing data was high, and the obtained transcripts were reliable and could be used for subsequent bioinformatics analysis. The original data of this study were submitted to the NCBI database with accession number PRJNA1231595. The datasets contained functional annotations for 34,648 genes in total. The genes that were differently expressed between R, B, 2R1B, and 4R1B and CK were chosen based on the criteria of *p*-adjust < 0.05 and |log2FC| ≥ 1. A total of 112 differentially expressed genes (DEGs) (52 up-regulated genes and 60 down-regulated genes) were identified in R (Figure S1A). For B, 153 DEGs were identified, with 65 genes being up-regulated and 88 down-regulated (Figure S1B). Moreover, 86 (51 up- and 35 down-regulated) and 251 (58 up- and 193 down-regulated) DEGs were identified in the 2R1B (Figure S1C) and 4R1B (Figure S1D), respectively.

**Figure 6:**
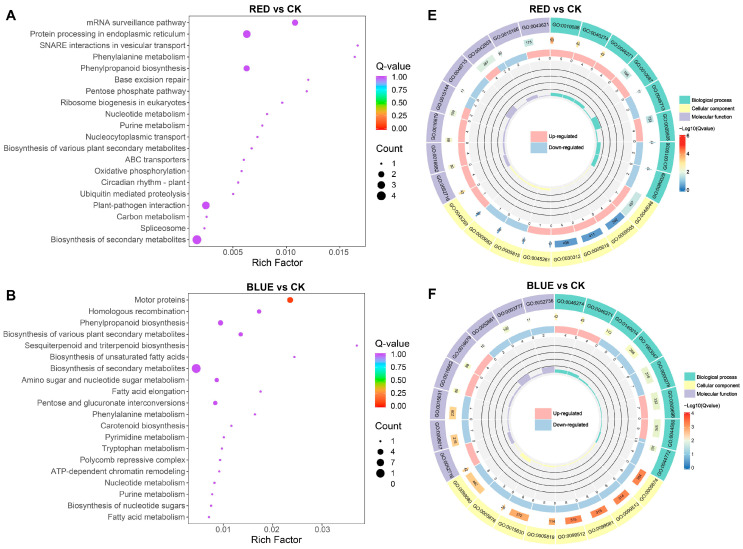

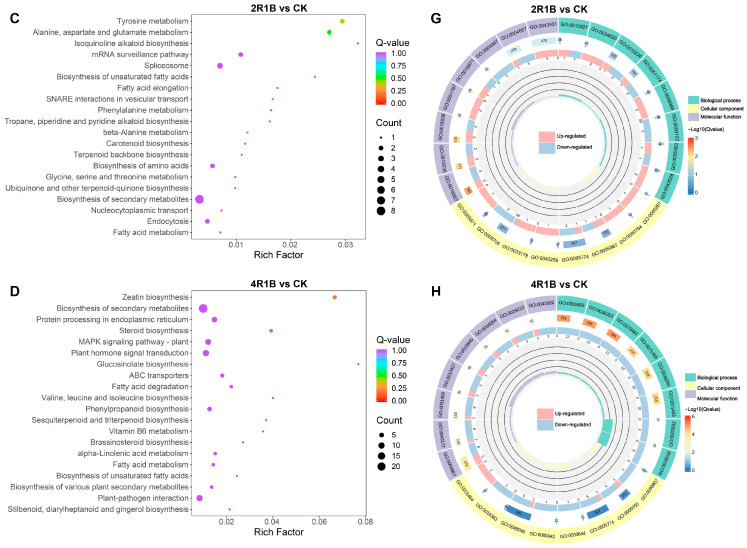
(**A**–**D**) Bubble diagram of KEGG enrichment results of different genes in different light treatments. The abscissa is the Rich factor. The larger the Rich factor, the greater the degree of enrichment, and the ordinate represents the KEGG pathway. The larger the dots, the greater the number of differentially expressed genes enriched in the pathway. The redder the dot, the more significant the enrichment. (**E**–**H**) GO enrichment circle diagram of differentially expressed genes in different light treatments. From the outside to the inside, the first circle is the three GO categories, and different colors represent different GO categories. Second circle: the number of the category in the background genes and the q-value. The more genes, the longer the bar, and the more significant the enrichment, the more red. Third circle: bar graph of the proportion of up-regulated and down-regulated genes, where light red represents the proportion of up-regulated genes, and light blue represents the proportion of down-regulated genes. Fourth circle: Rich factor values for each category.

According to KEGG enrichment analysis, DEGs were significantly enriched in metabolic pathways, including biosynthesis of secondary metabolites, in the four comparison groups (Figure 6A–D and Figure S1E–H). Moreover, 4R1B treatment positively enriched MAPK signaling pathway–plant, plant hormone signal transduction, and plant–pathogen interaction (Figure S1H). To further reveal the biological functions of DEGs in *G. uralensis* under light treatment, the DEGs were divided into three main functional categories using GO analysis: Biological Process (BP), Molecular Function (MF), and Cell Component (CC) (Figure S1I–L). The DEGs of R vs. CK, B vs. CK, 2R1B vs. CK, and 4R1B vs. CK were significantly enriched in 557, 792, 525, and 863 GO terms, respectively (Figure 6E–H). In the four pairwise comparisons, the top five terms in the CC category were supramolecular fiber (GO:0099080), supramolecular polymer (GO:0099081), polymeric cytoskeletal fiber (GO:0099513), microtubule (GO:0015630), and apoplast (GO:0048046). In the BP category, the most enriched terms were response to oxygen levels (GO:0070482), response to decreased oxygen levels (GO:0036294), response to hypoxia (GO:0001666), response to oxidative stress (GO:0006979), cellular response to oxygen levels (GO:0071453), and cellular response to hypoxia (GO:0071456). In the Molecular Function category, enzyme inhibitor activity (GO:0004857), lipid binding (GO:0008289), carboxylic acid binding (GO:0031406), organic acid binding (GO:0043177), monooxygenase activity (GO:0004497), tubulin binding (GO:0015631), and microtubule binding (GO:0008017) were the most highly represented terms.

### 2.7. Integrated Analysis of Genes and Metabolites of G. uralensis Treated with Different Lights

KEGG enrichment analysis of DEGs and DAMs screened in the transcriptome and metabolome in the different light treatment groups and the CK group was performed. The results showed that they were mainly co-enriched in two pathways, namely metabolic pathways and biosynthesis of secondary metabolites (Figure S2). Moreover, the DEGs of the blue light group and the CK group were significantly enriched in flavonoid biosynthesis, and the DAMs of the 4R1B group and the CK group were significantly enriched in plant hormone signal transformation. Based on this, we analyzed the metabolic pathways of the main active components of *G. uralensis* (liquiritin and glycyrrhizic acid) and plant hormones.

#### 2.7.1. DEG Analysis of Flavonoid and Terpenoid Synthesis Pathways

**Figure 7:**
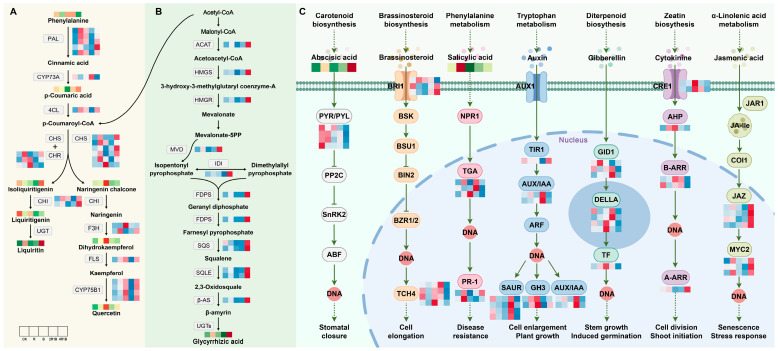
Expression changes of key enzyme genes for flavonoid synthesis pathway in different light treatments. All arrows indicate direction of biosynthesis pathway. Solid, dotted and T arrows refer to direct generation, indirect generation, and inhibition respectively. (**A**) Flavonoid biosynthesis pathway in *G. uralensis*. (**B**) Terpenoid biosynthesis pathway in *G. uralensis*. (**C**) Plant hormone signal transduction pathway in *G. uralensis*.

The main flavonoid in *G. uralensis* is liquiritin. To analyze the potential reasons for the changes in the flavonoid contents of *G. uralensis* treated with the different lights, we identified the transcription levels of nine enzyme genes that participated in flavonoid biosynthesis. The putative network of flavonoid biosynthesis is displayed in Figure 7A. The *PAL* genes are involved in the process of phenylalanine forming cinnamic acid, and most genes were up-regulated under the R, B, and 4R1B treatments. The *CYP73A* and *4CL* genes catalyze cinnamic acid to form *p*-coumaroyl-CoA, while the expression patterns of these two genes are opposite. *CYP73A* was significantly up-regulated under the 2R1B treatment, and *4CL* was up-regulated under the R, B, and 4R1B treatments. The *CHS* and *CHR* genes catalyze *p*-coumaroyl-CoA to transform into isoliquiritigenin, and isoliquiritigenin is then converted into liquiritigenin through the *CHI* gene. These genes showed similar expression profiles, exhibiting higher expression levels in the R-, B-, and 4R1B-treated groups than in the CK group. HPLC analysis of *G. uralensis* extract determined that the liquiritin contents of all light treatments were higher than that of the CK group. The content of the 4R1B treatment and R treatment was 3.75 and 2.91 times that of the CK group, respectively. These results suggest that red light- and 4R1B-induced metabolic flux of liquiritin in *G. uralensis* relies on several key steps, namely: (1) conversion of phenylalanine to cinnamic acid; (2) conversion of *p*-coumaric acid to *p*-coumaroyl-CoA; and (3) conversion of *p*-coumaroyl-CoA to isoliquiritigenin.

In this study, we explored the effects of different light treatments on the accumulation of terpenoid metabolites by screening candidate genes and metabolites involved in *G. uralensis* terpenoid synthesis or modification. A putative triterpenoid biosynthesis network revealed that treatments with various light types affected the precursor steps of glycyrrhizic acid biosynthesis (Figure 7B). Glycyrrhizic acid is synthesized through the methyl valproic acid (MVA) pathway, with the starting ingredient being acetyl-CoA [42]. Triterpene saponins are derived from terpenoid backbone biosynthesis, and *ACAT*, *HMGS*, and *HMGR* are important functional genes associated with terpenoid backbone synthesis. As shown in Figure 7B, all three genes had similar expression patterns, showing significant up-regulation in the 4R1B group. The process created isopentenyl pyrophosphate and dimethylallyl pyrophosphate, which are utilized as precursors to create terpenoid metabolites under the influence of *MVD* and *IDI*. Especially, the genes involved in the subsequent synthesis of the glycyrrhetinic acid pathway (*FDPS*, *SQS*, *SQLE*, *β-AS*) were all significantly highly expressed in the 4R1B group. The determination results showed that the 4R1B group and the R group had significantly increased glycyrrhetinic acid content, which was 1.94 times and 1.48 times greater than that of the CK group.

Triterpenoids and flavonoids are important markers of *G. uralensis* quality. We observed an increase in the gene expression of most flavonoids in *G. uralensis* in response to the red, blue, and 4R1B mixed light conditions. The enzymes *PAL*, *C4H*, and *4CL* play important roles upstream in the flavonoid biosynthesis pathway. An increase in the expression of *PAL*, *C4H*, and *4CL* could lead to an increase in the precursor substances (like p-coumaric acid) involved in flavonoid synthesis. *CHI* and *CHS* are the key enzymes in the synthesis pathway of liquiritin in *G. uralensis* [39,40], which corroborates the high liquiritin content of *G. uralensis* in the 4R1B group and the red group. Moreover, *CHR*, *CHI*, *F3H*, and *FLS* are essential branch-point genes that regulate flavonoid accumulation [43]. In this study, the content of some flavonoids (naringenin chalcone, dihydrokaempferol, and quercetin) was positively correlated with the relative expression of *PAL*, *4CL*, and *CHS* in *G. uralensis*. Similar results were reported in *Cyclocarya paliurus* [44]. Interestingly, the expression of most phenylpropanoid and flavonoid pathway genes in strawberry were reduced under monochromatic red or blue light as compared to white light [43], suggesting that the mechanism for red/blue light-dependent flavonoid accumulation is not universal across all plant species.

Glycyrrhizic acid is a triterpene saponin with unique biological activities in *G. uralensis* [1], and its biosynthesis is initiated from the MVA pathway of the terpene backbone. We discovered that the gene expression of structural triterpenoids, including *ACAT*, *HMGS*, *HMGR*, *MVD*, *IDI*, *FDPS*, *SQS*, and *β-AS*, increased substantially in the 4R1B group. Meanwhile, the accumulation of glycyrrhizic acid in 4R1B was significantly higher than that in the other treatment groups. Wang et al. [45] showed that light, especially blue and red light, plays an important regulatory role in the synthesis and accumulation of plant secondary metabolites. Moreover, the expression of the salvianolic acid synthesis genes *PAL* and *4CL* is induced by red and blue light [24]. Kubica et al. [46] reported that the isoflavone glycoside content of *Verbena offcinalis* was significantly increased under red, blue, and 3B:7R light. The anthocyanin content was increased significantly in *Pfaffa glomerata* treated with 1B:1R light [47]. The light conditions promoting accumulation differ among metabolites due to these compounds playing different roles in the response to environmental factors [48].

#### 2.7.2. DEG Analysis of Plant Hormone Signaling Pathways

It is well known that phytohormones have a critical impact on plant growth and development [49]. Under different light treatments, we identified seven phytohormone signaling pathways, including abscisic acid, brassinosteroid, salicylic acid, auxin, gibberellin, cytokinin, and jasmonic acid, which were associated with 19 DEGs in *G. uralensis* (Figure 7C). In the abscisic acid (ABA) signaling pathway, the 4R1B group had the highest ABA content. In contrast, the *PYR/PYL* genes were significantly highly expressed in the CK and R groups. Previous studies have shown that *TCH4* is associated with the regulation of plant cell elongation. Figure 7C shows that the *TCH4* gene was significantly down-regulated in both the R and 4R1B treatment groups. Bergonci et al. [50] found through *Arabidopsis* mutants that the *TCH4* genes in plants with long root lengths and long cotyledons were down-regulated. This result reveals why the length of *G. uralensis* in the R and 4R1B groups was significantly greater than in the other groups. The R group had the highest salicylic acid (SA) content. Under the action of SA, NPR1 stimulates the DNA binding activity of interacting TGA factors, which then bind to SA response elements in response to certain biotrophic pathogens [51,52,53]. Moreover, *PR-1* gene expression is modulated in plant tissues by defense-related signaling molecules, the expression of which depends on SA [54]. The results showed that two transcripts of the *PR-1* gene were up-regulated in the R group, and one transcript was up-regulated in the 4R1B group. Fang et al. [55] found that the overexpression of *PR-1* enhanced plant resistance to Morus multicaulis. This result may indicate that red light is more conducive to improving the disease resistance of *G. uralensis* plants. The plant hormone indole-3-acetic acid (IAA or auxin) regulates many aspects of plant growth and development, including stem elongation and the lateral branching of roots and shoots [56,57]. Auxin-mediated changes in cell division, expansion, and differentiation control these processes. Figure 7C shows the expression was highest in the 4R1B group, a result consistent with the *G. uralensis* seedlings in the 4R1B group in this study having the highest height. Recent studies have revealed that the gibberellin (GA) receptors *GID1*, *GID2*, *DELLA*, and the *F-box* proteins, participate in GA signaling in both Arabidopsis and rice. *GID1* binds bioactive GAs to promote the formation of the *GID1-DELLA* complex by interacting with the N-terminal domain of *DELLAs* [58]. The results showed that the expression patterns of DEGs under the GA pathway were inconsistent. Overall, in the R, B, and 2R1B groups, gene expression was up-regulated. Jasmonic acid (JA) and its conjugate form, JA-Ile, have been implicated in the responses to abiotic challenges and biotic threats [59,60,61]. In *Arabidopsis*, *MYC2* regulates wounding-induced JA accumulation by directly binding to the promoters of genes functioning in JA biosynthesis and catabolism to promote their transcription. The expression of the *JAZ* and *MYC2* genes was significantly increased in the 4R1B group, suggesting that the 4R1B treatment group may have stronger resistance to biotic and abiotic stresses.

## 3. Materials and Methods

### 3.1. Plant Material and Growth Conditions

*G. uralensis* seeds were collected from Yanchi County, Ningxia Province, China. The seeds of *G. uralensis* were treated with 98% concentrated H_2_SO_4_ for 30 min and washed with sterilized distilled water three times. The germinated seedlings were cultured in a growth chamber under the following conditions: 50 μmol m^−2^·s^−1^ white light, 16 h light, 24 °C; 8 h dark, 18 °C, and relative humidity 50%. After 2 months, they were subjected to light treatments.

### 3.2. Light Treatments

We utilized light-emitting diode (LED) (ZPDT802-60, Beijing Shengyanggu Technology Co. LTD, Beijing, China) lamp beads as a light source in the artificial-climate laboratory. The control group was treated with white light (300–800 nm), designed blue light (460 nm), and red light (660 nm). The different red and blue light combinations were evenly cross-arranged according to red/blue = 2:1/4:1, and light intensity was controlled to 4700 ± 300 lx. According to the ratio of the number of LED lamp beads (red to blue light), the experiments included the following groups: white light (CK), red light (R), 4R1B (red/blue = 4:1), 2R1B (red/blue = 2:1), and blue light (B). The culturing conditions including light intensity, photoperiod, temperature, and relative humidity were identical to those in Section 3.1. After 30 days of treatment, for RNA sequencing and targeted metabolomics, ten fresh roots of *G. uralensis* were mixed as a sample, and three replicates were carried out. All fresh samples for biochemical analysis were frozen in liquid nitrogen immediately and then stored in a freezer at −80 °C.

### 3.3. Measurements of Photosynthetic Rate and Chlorophyll Concentration

We used Yaxin-1105 Portable Photosynthetic Fluorometer (Beijing, China) to measure the net photosynthetic rate (Pn) and stomatal conductance (Gs) of five healthy leaves of *G. uralensis* per treatment. The measurement time was 9:00 am to 11:00 am, and the CO_2_ concentration was set at 400 μmol·mol^−1^ and the air flow rate at 0.6 m·min^−1^.

The contents of chlorophyll a, chlorophyll b, and carotenoids in leaves were determined as described previously [62,63]. Briefly, 0.1 g fresh healthy leaves of *G. uralensis* seedlings were weighed, added to 10 mL 95% ethanol, and stored in the dark for 3 h after grinding. The absorbance at 665, 649, and 470 nm was determined, and the chlorophyll a, chlorophyll b, total chlorophyll, and carotenoid content was calculated with reference to the plant chloroplast pigment assay kit instructions (Jiangsu Aidisheng Biological Technology Co., Ltd., Yancheng, China).

### 3.4. Physiology, Biochemistry, and Medicinal Component Analysis

After 2 months of different light treatments, five *G. uralensis* seedlings were randomly selected for each treatment, and the morphological indices were determined for five parallel repetitions. The plant high, root length, dry shoot weight, dry root weight, and diameter of reed head were measured. The contents of soluble protein and soluble sugar were determined by the Coomassie brilliant blue method and anthrone colorimetry method, respectively [64,65,66]. The quantification of photosynthetic pigments was executed by employing the method outlined by Asghar et al. [67]. The activities of SOD and MDA were analyzed according to the method described by Fan et al. [68]. The contents of glycyrrhizic acid and liquiritin were detected according to the method of CHP [9].

### 3.5. Targeted Metabolomic Analysis

Fresh samples were freeze-dried, ground into powder, and extracted with 70% methanol solution. The supernatant was taken and filtered through a 0.22 mm pore-size membrane filter and stored in a chromatography sample vial. The sample extracts were analyzed using an UPLC-ESI-MS/MS system (UPLC, ExionLC™ AD, Shanghai, China) and tandem mass spectrometry system. The analytical conditions were as follows. UPLC: column, Agilent SB-C18 (1.8 µm, 2.1 mm × 100 mm); mobile phase: pure water with 0.1% formic acid (solvent A), acetonitrile with 0.1% formic acid (solvent B). The gradient elution concentration was: 5% B (0 min); 95% B (9 min); 95% B (1 min); 5% B (0.1 min); 5% B (2.9 min). All separations were carried out at a constant column temperature of 40 °C under a 360 nm wavelength and flow rate of 0.35 mL/min. The injection volume was 2 μL. A triple quadrupole linear ion trap mass spectrometer (Q TRAP) with an ESI Turbo Ion-Spray interface on an AB Sciex QTRAP 4500 System, operated by Analyst 1.6.1 software, was used to obtain LIT and QQQ scans. The operating parameters of the ESI source were as previously described [69].

MultiaQuant was used to process the amount and quality of metabolites using a local Metware Biotechnology metabolite database. The best-differentiated metabolites between treatments were filtered using the applied OPLS-DA model’s variable importance of projection (VIP) score. For two-group analysis, differential metabolites were determined by VIP (VIP > 1) and absolute log2FC (|log2FC| ≥ 1.0). KEGG pathways were used to functionally annotate differentially accumulated metabolites (DAMs).

### 3.6. Transcriptome Sequencing and Bioinformatics Analysis

The total RNA from *G. uralensis* seedling roots (CK, R, B, 4R1B, and 2R1B) was extracted by RNeasy Pure Plant Kit (DP432, Tiangen, China). The extracted RNA was electrophoresed on a 1% agarose gel and detected by a NanoPhotometer spectrophotometer (IMPLEN, Los Angeles, CA, USA). Then, double-stranded cDNA preparation was performed using the RNA Library Prep Kit (Tiangen Bio, Beijing, China). After amplification and purification, the cDNA libraries were sequenced using the Illumina HiSeq platform (Illumina Inc., San Diego, CA, USA) provided by Metware Biotechnology Co., Ltd. (Wuhan, China). The original data of this study were submitted to the NCBI database with accession number PRJNA1231595. The raw data were filtered, checked for sequencing error rates, and checked for GC content distribution to obtain clean reads. After obtaining clean reads, Trinity assembly was used to splice them to obtain reference sequences for subsequent analysis [70]. Taking the transcript sequence assembled by Trinity as a reference, unigenes of each sample were obtained through Corset hierarchical clustering.

The unigene sequences were aligned with the Kyoto encyclopedia of genes and genomes (KEGG), non-redundant (NR), SWISS-PROT, gene ontology (GO), EuKaryotic Orthologous Groups (KOG), and Pfam databases to obtain the annotation information of unigenes [71]. Sequencing reads were compared to unigene library using Bowtie, and expression levels were estimated with RSEM software (http://deweylab.biostat.wisc.edu/rsem/; accessed on 12 October 2023) [72]. The determination of gene expression levels was conducted in accordance with the FPKM values. The read count was normalized, and DESeq2 was used to determine the differentially expressed genes (DEGs) between A and B. The screening threshold for DEGs was |log2 Fold Change| ≥ 1 and FDR < 0.05 [73]. The topGO approach, which is based on the Wallenius non-central hypergeometric distribution, was used to conduct the GO enrichment analysis. KOBAS2.0 was used to conduct the KEGG pathway enrichment analysis of the DEGs.

### 3.7. Co-Expression Analysis

The values were expressed as the mean values, with the error indicated by the standard deviation with three biological replicates. Data were analyzed using one-way (ANOVA) and Duncan’s test in the SPSS 27.0 software and presented as means ± standard deviation, and statistical significance was considered at *p* < 0.05. The figures were generated using GraphPad Prism 10, Adobe Photoshop 2020, and Adobe Illustrator CC 2019. The transcriptome and metabolome data were log-transformed (log2) for integration analysis. The differential genes and metabolites were mapped onto the KEGG pathways at the same time. Hierarchical clustering analysis (HCA), orthogonal partial least discriminant squares (OPLS-DA) analysis, and maps obtained from R software (www.r-project.org; accessed on 16 October 2023.) were used to study DEMs accession-specific accumulation.

## 4. Conclusions

The combination of red and blue light can affect plant growth, physiology, and the biosynthesis of secondary metabolites. According to the growth indicators, red light could promote the growth of *G. uralensis* seedlings, such as the plant height, root length, diameter of the reed head, and whole-plant biomass accumulation. Blue light inhibited the reed head diameter, whole-plant biomass accumulation, and root–-shoot ratio. Regarding the mixed light, 4R1B with more red light could promote the growth of *G. uralensis* seedlings, such as the root length, diameter of the reed head, root dry weight, and root–shoot ratio. The influences of different light spectra on the photosystem were reflected in blue light significantly inhibiting the photosynthetic rate and stomatal conductance, while red light and mixed light increased the photosynthetic rate and stomatal conductance in 60–90 d. In addition, photosynthetic pigment production was inhibited during 30–60 d of light treatment but promoted during 90 d of light treatment in the 2R1B group. The red light enhanced the antioxidant enzyme activity in *G. uralensis*, removing reactive oxygen species and promoting the synthesis of soluble sugars and soluble proteins. Combined UPLC-MS/MS-based metabolomics and transcriptomics analysis revealed the underlying biological mechanism responsible for the different light spectral-induced increases in liquiritin and glycyrrhizic acid contents in cultured *G. uralensis*. The results showed that red, blue, and 4R1B mixed light could up-regulate the expression of genes in the glycyrrhizin synthesis pathway, such as *PAL*, *4CL*, *CHS*, and *CHI*. Blue light can promote the content of most flavonoids, and 4R1B can increase the accumulation of liquiritin content. 4R1B mixed light significantly promoted the expression of genes in the glycyrrhizic acid synthesis pathway, and the content of glycyrrhizic acid was significantly increased. In addition, different light spectra affected seven phytohormone pathways of *G. uralensis*, which can promote cell elongation, stem elongation, stress resistance, and other aspects. In general, we analyzed the response of *G. uralensis* to combined red and blue light at the physiological, medicinal component, and molecular levels, which will provide a new perspective on the response of plants to combined red and blue light. Our findings also provide invaluable data for delving into the molecular and chemical mechanisms that drive the flavonoid and terpenoid biosynthesis pathways in Glycyrrhiza plants when exposed to various light spectra.

## Data Availability

All sequences used in this study are available in the form of attachments. The original contributions presented in the study are publicly available. The data can be found here: https://www.ncbi.nlm.nih.gov/bioproject/PRJNA1231595; access on 6 March 2025.

## Supplementary Materials

Supporting information can be downloaded at https://www.mdpi.com/article/10.3390/ijms26104641/s1.

## Abbreviations

The following abbreviations are used in this manuscript:

CHP	Pharmacopoeia of the People’s Republic of China
NCBI	National Center for Biotechnology-Information
DAMs	Differentially accumulated metabolites
DEGs	Differentially expressed genes
MVA	methyl valproic acid
