# Supplementary figures and images for: Transcriptome and Metabolomics Analysis Reveal the Effects of Red and Blue Light on the Physiology and Primary Medicinal Components (Liquiritin and Glycyrrhizic Acid) of Glycyrrhiza uralensis Seedlings
Source: Int J Mol Sci. 2025 May 13;26(10):4641. doi: 10.3390/ijms26104641 (PMC12110894; doi:10.3390/ijms26104641)

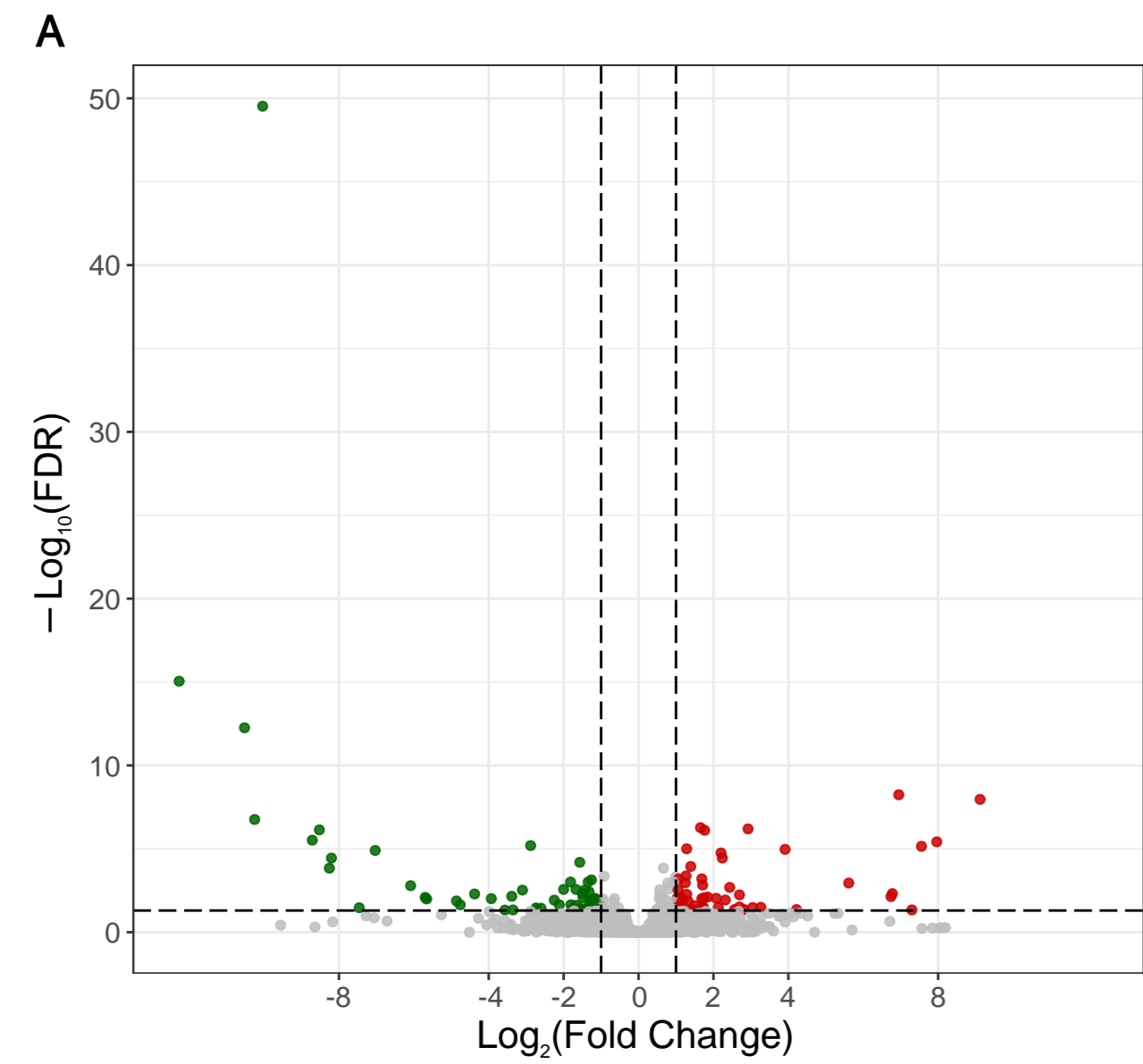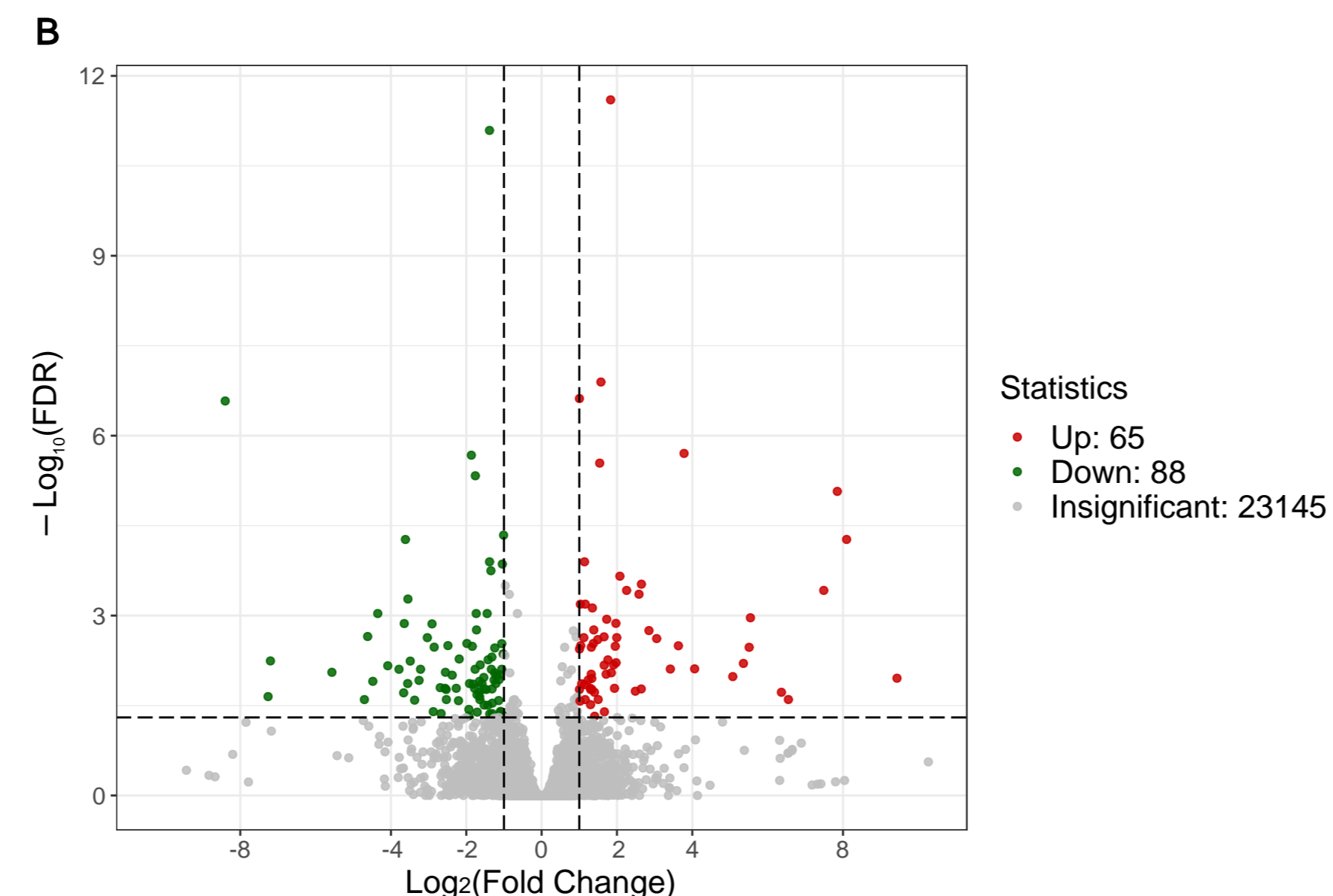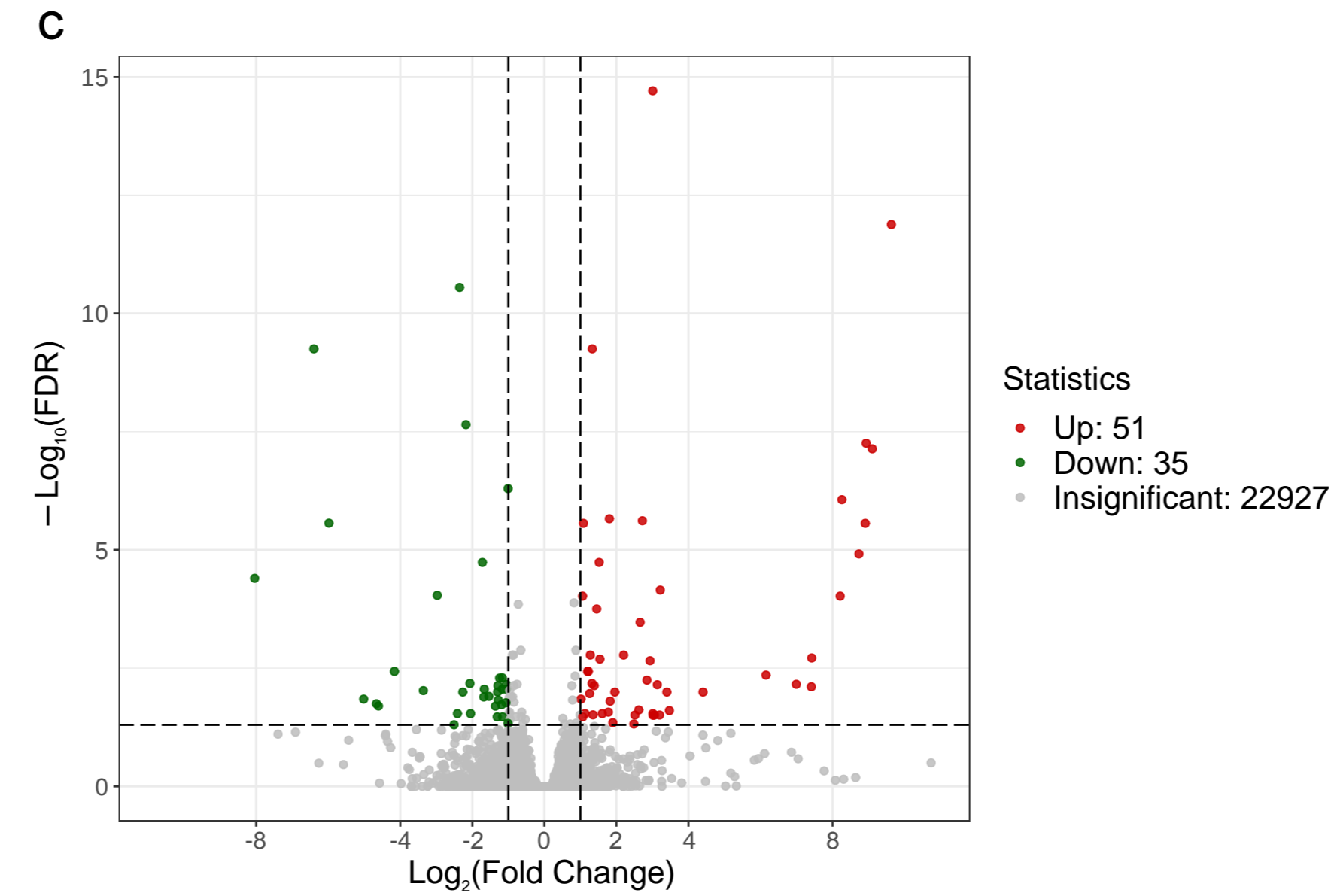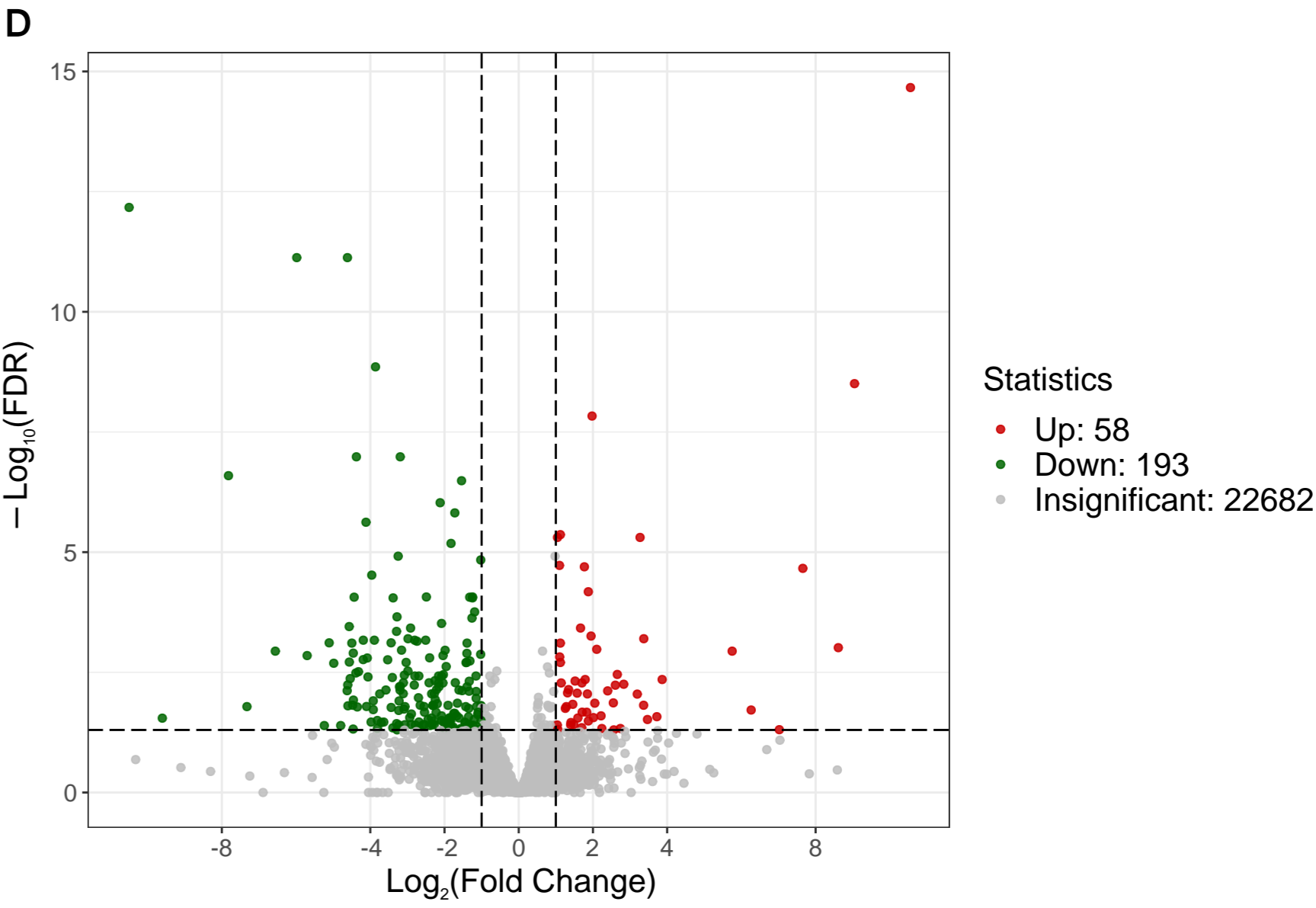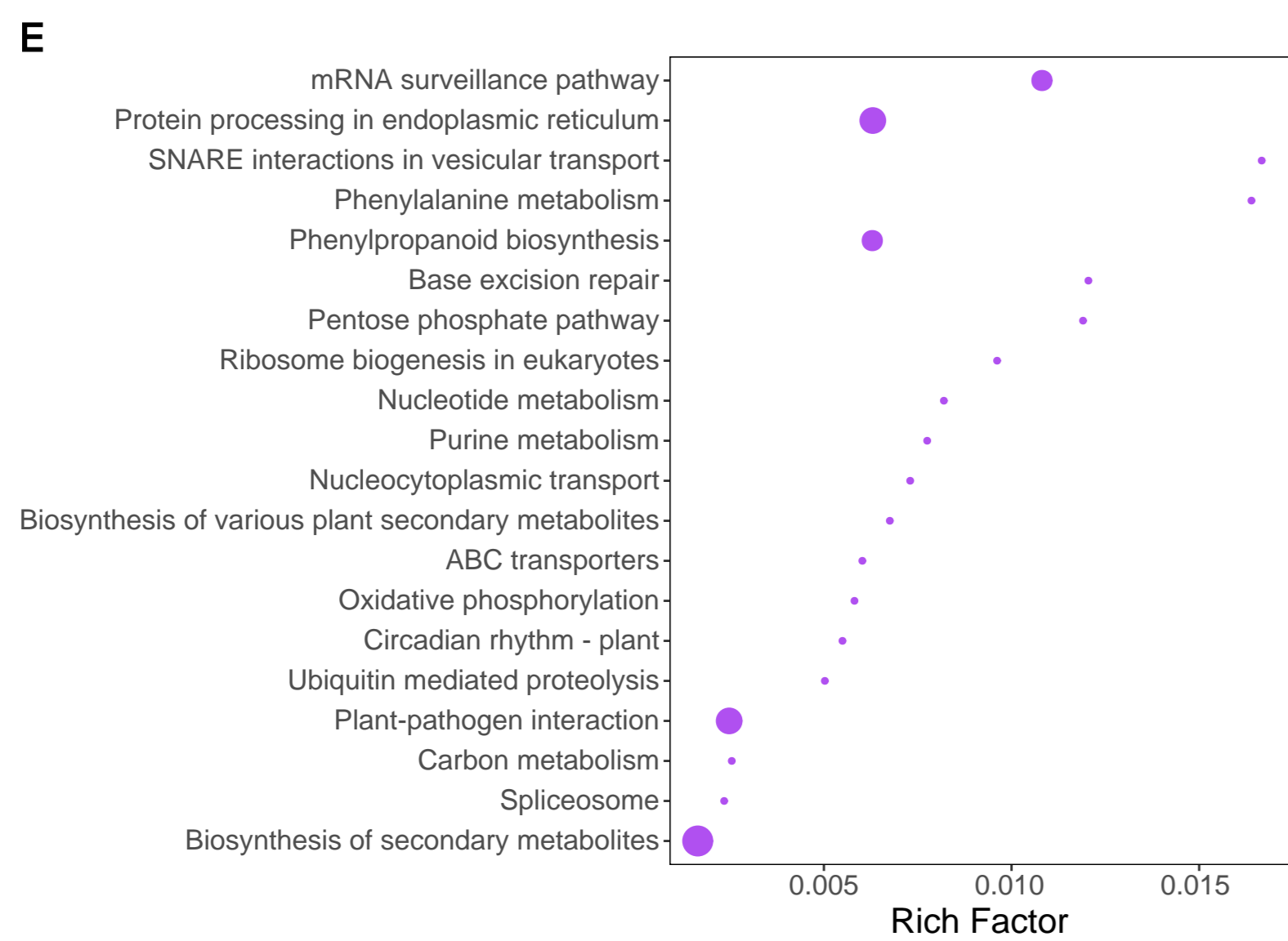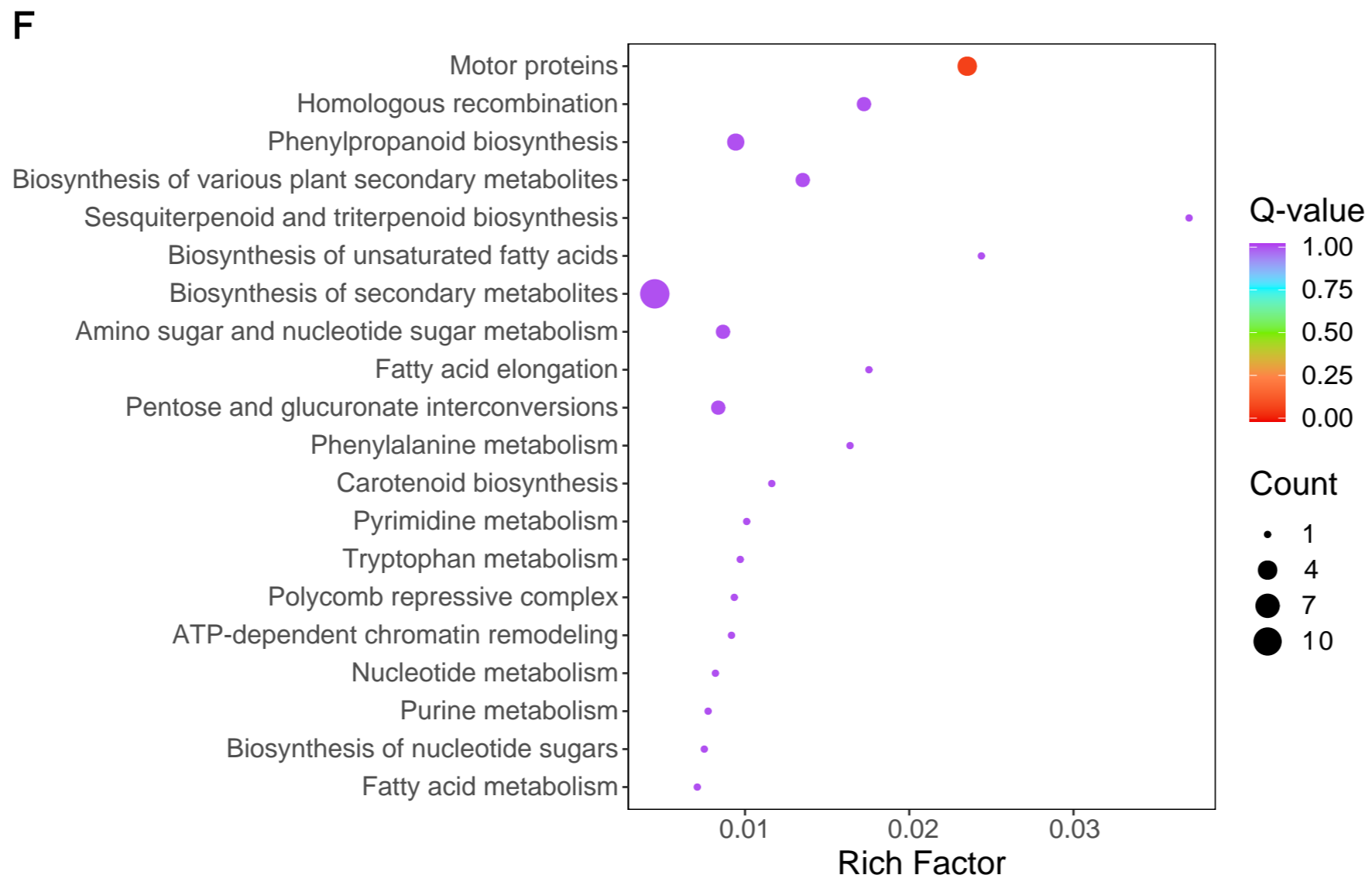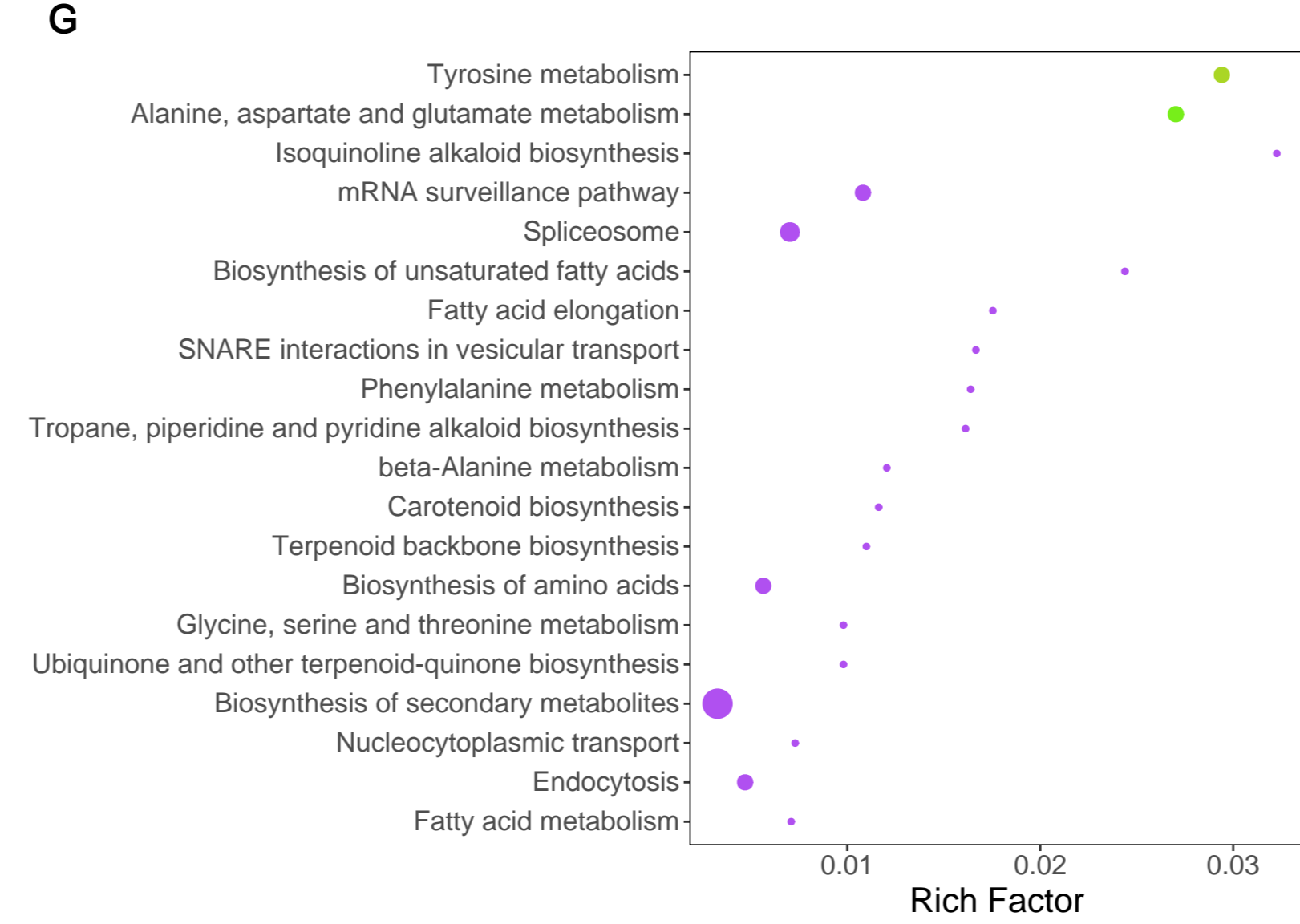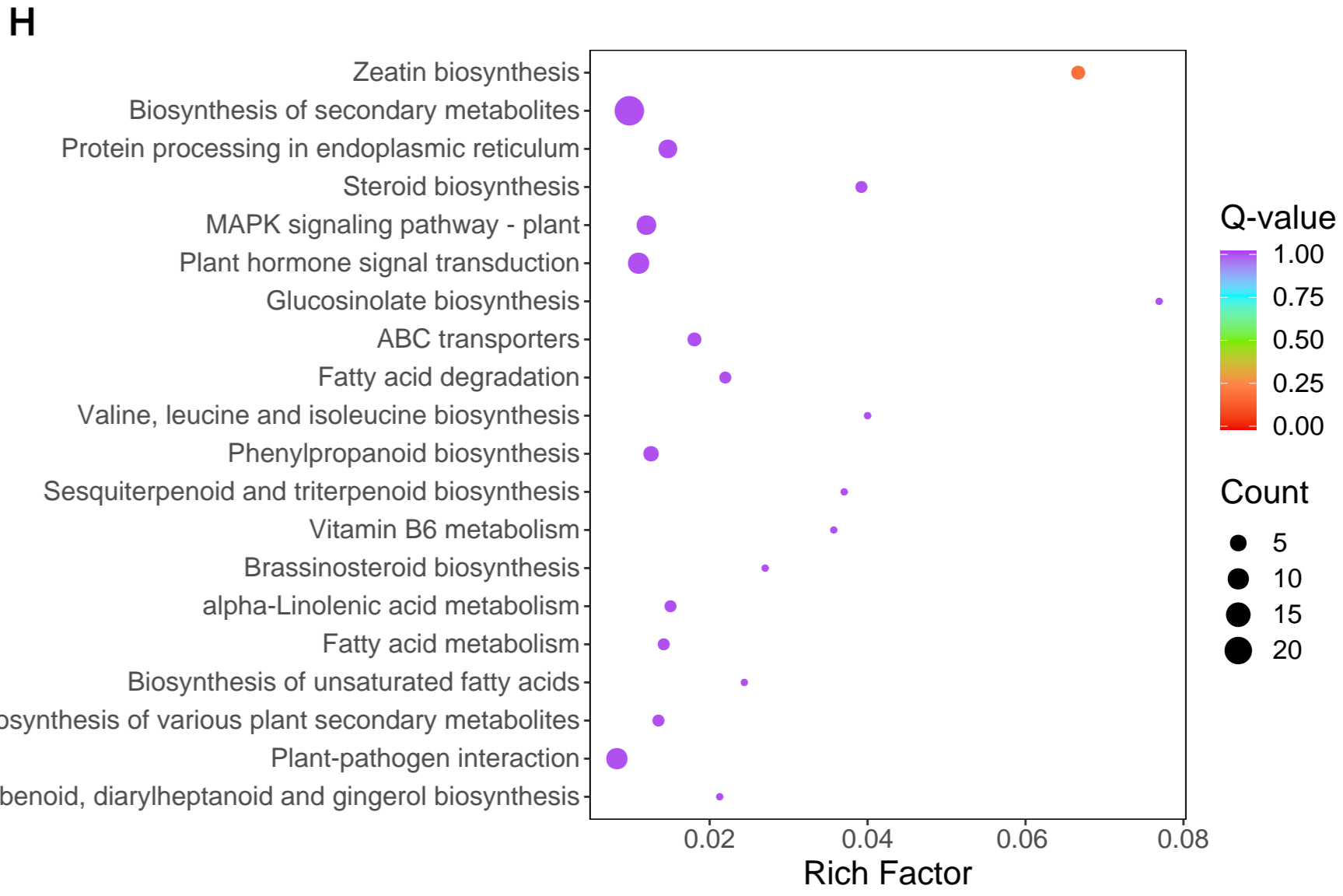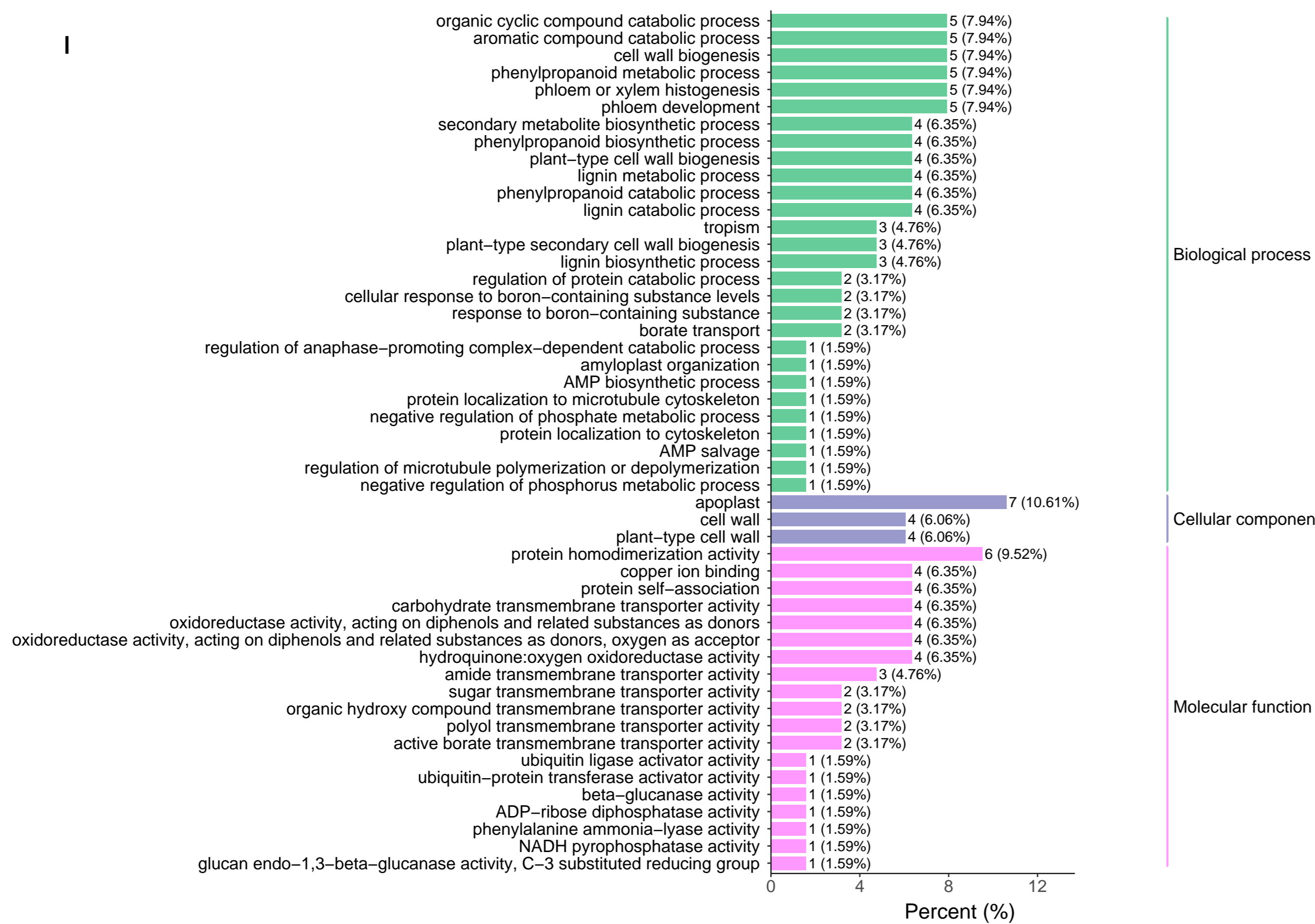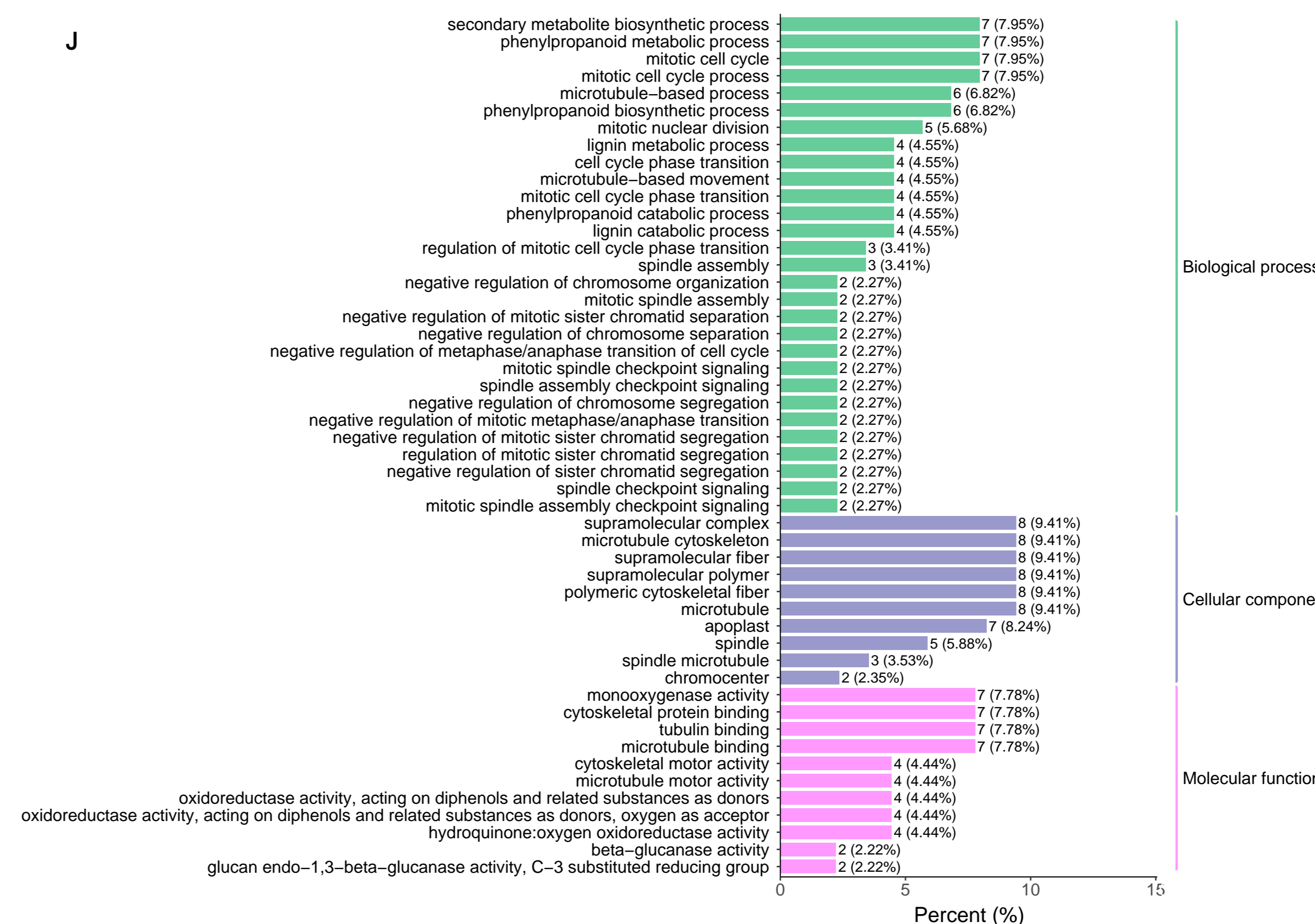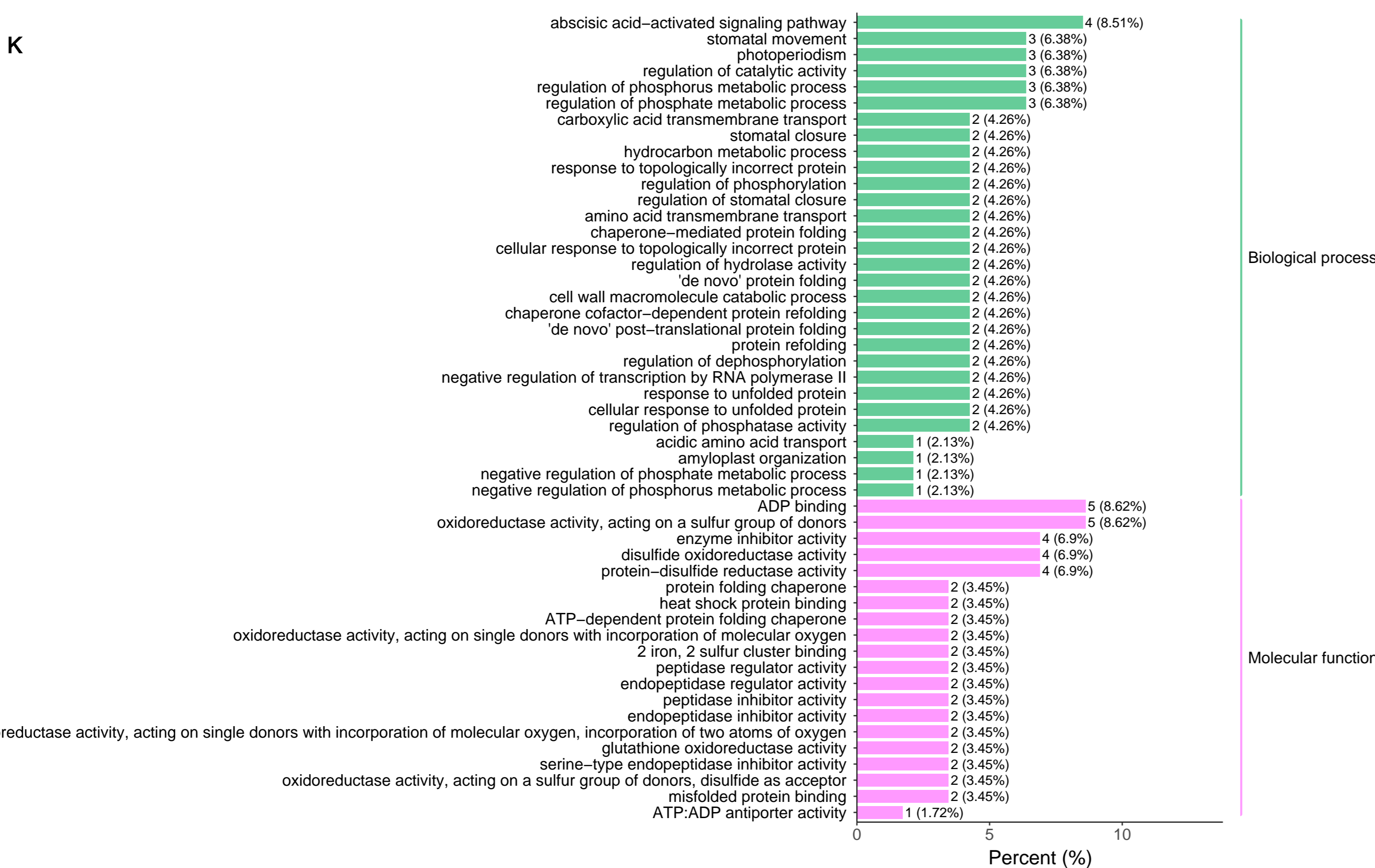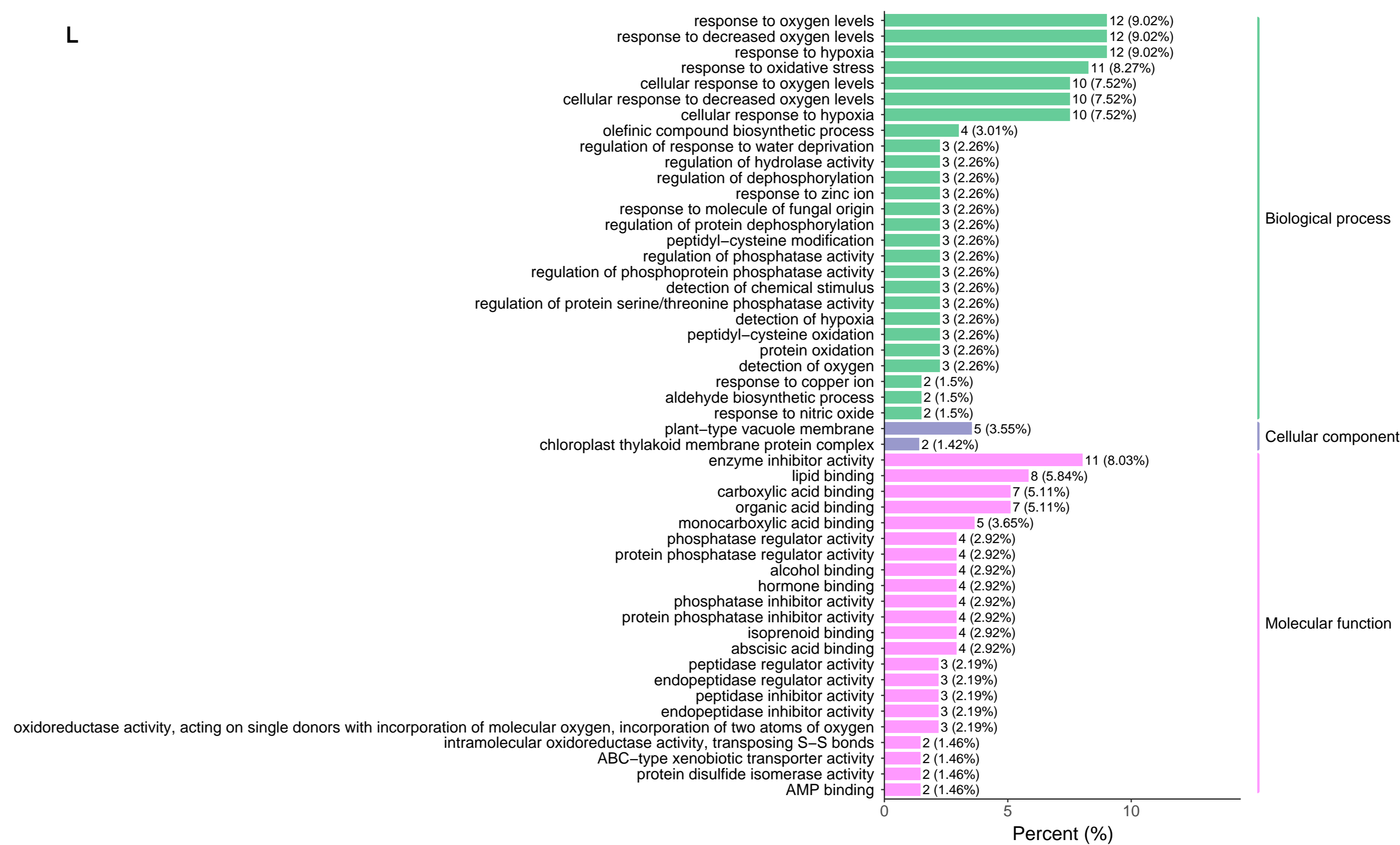

Supplement: Supplementary file 1 [file ijms-26-04641-s001.zip › Figure S1.pdf]

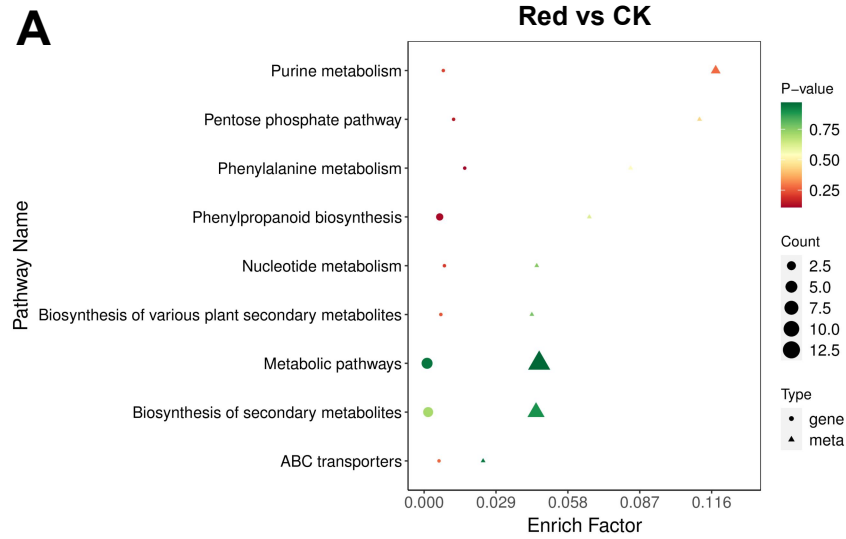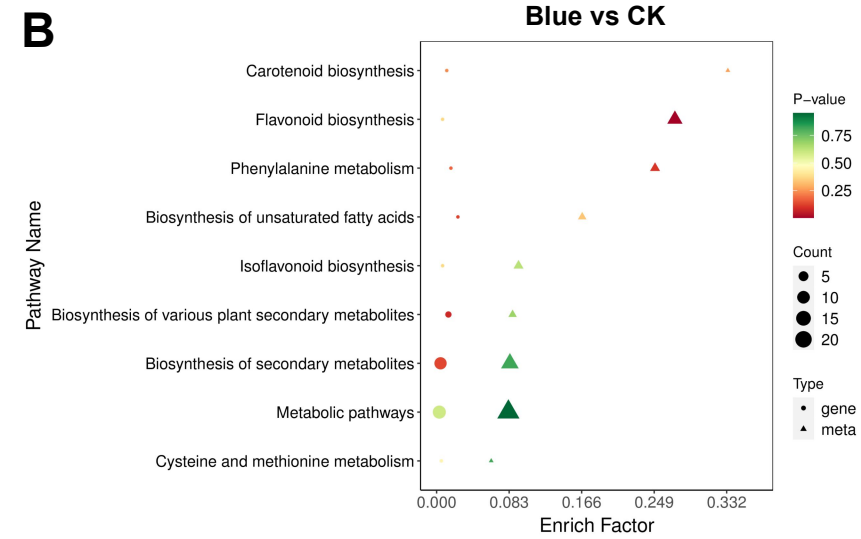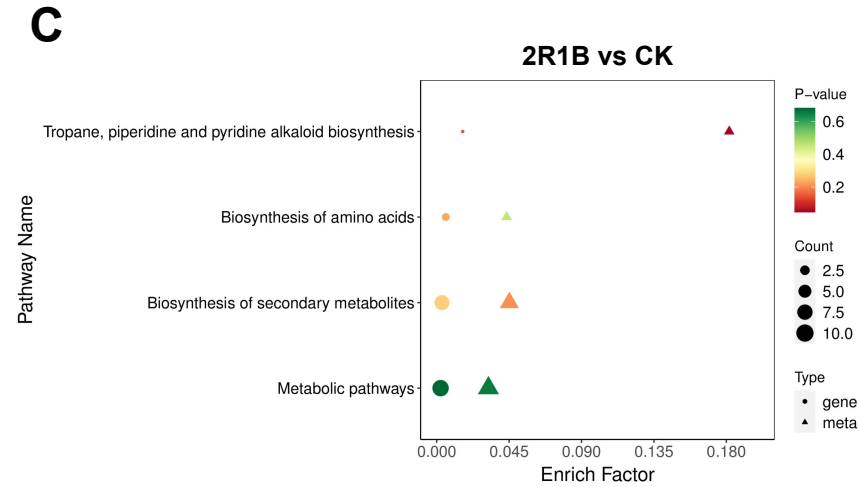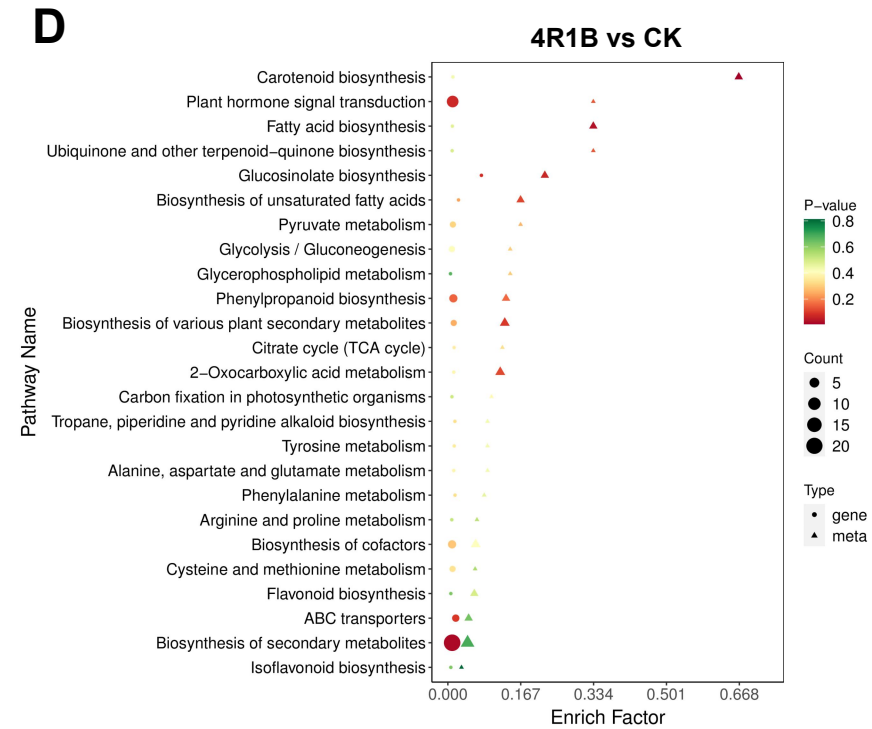

Supplement: Supplementary file 1 [file ijms-26-04641-s001.zip › Figure S2.pdf]
